# Nanoparticles: Health Effects—Pros and Cons

**DOI:** 10.1289/ehp.8871

**Published:** 2006-08-18

**Authors:** Maureen R. Gwinn, Val Vallyathan

**Affiliations:** National Institute for Occupational Safety and Health, Morgantown, West Virginia, USA

**Keywords:** cons, nanoparticle toxicity, nanotechnology, pros

## Abstract

With the advent of nanotechnology, the prospects for using engineered nanomaterials with diameters of < 100 nm in industrial applications, medical imaging, disease diagnoses, drug delivery, cancer treatment, gene therapy, and other areas have progressed rapidly. The potential for nanoparticles (NPs) in these areas is infinite, with novel new applications constantly being explored. The possible toxic health effects of these NPs associated with human exposure are unknown. Many fine particles generally considered “nuisance dusts” are likely to acquire unique surface properties when engineered to nanosize and may exhibit toxic biological effects. Consequently, the nuisance dust may be transported to distant sites and could induce adverse health effects. In addition the beneficial uses of NPs in drug delivery, cancer treatment, and gene therapy may cause unintentional human exposure. Because of our lack of knowledge about the health effects associated with NP exposure, we have an ethical duty to take precautionary measures regarding their use. In this review we highlight the possible toxic human health effects that can result from exposure to ultrafine particles (UFPs) generated by anthropogenic activities and their cardiopulmonary outcomes. The comparability of engineered NPs to UFPs suggests that the human health effects are likely to be similar. Therefore, it is prudent to elucidate their toxicologic effect to minimize occupational and environmental exposure. Highlighting the human health outcomes caused by UFPs is not intended to give a lesser importance to either the unprecedented technologic and industrial rewards of the nanotechnology or their beneficial human uses.

The advent of nanotechnology is considered to be the biggest engineering innovation since the Industrial Revolution. Proponents of this new technology promise to reengineer the man-made world, molecule by molecule, sparking a wave of novel revolutionary commercial products from machines to medicine (Aston 2005). This “industrial revolution” in molecular manufacturing will alter the relationship of materials so profoundly that this change may produce both positive and negative effects on health and the environment. The worldwide market for products produced using nanotechnology is estimated to reach US$1 trillion by 2015 (Roco 2005). The technologic progress during the Industrial Revolution enhanced quality of life but also resulted in a human health burden. As in the case of asbestos with its decades of long latency that still remain, there are many legitimate concerns about the unknown human health consequences of nanomaterials. Nanotechnology, now at the leading edge of rapid development with many potential human health benefits, is perceived with apprehension for potential human health risks. Enhanced strength, durability, flexibility, performance, and inimitable physical properties associated with these materials has been exploited in a multitude of industries and treatment modalities including detection of tumors, targeted drug delivery, and prognostic visual monitoring of therapy. With these applications, unprecedented avenues of exposure to nanoparticles (NPs) in humans are likely. Ambient and workplace exposures in combination with other toxic agents may cause unpredictable adverse health effects. Failure to address these imminent human health issues in a cohesive and concerted manner by industry, academia, government, environmentalists, and scientists may lead to detrimental health effects caused by exposure to NPs.

In addition to occupational exposure, direct human exposures through medicinal applications and ambient air pollution are a major concern. Inhaled NPs may evade phagocytosis, cross cell membranes, and redistribute to other sites of the body, causing systemic health effects. Therefore, the unbridled growth and use of nanotechnology in medical and human health evaluations opens society to the possibility that NPs could become the “asbestos” of the 21st century. In this review we discuss briefly some of the future human benefits of nanotechnology (pros) and emphasize possible health concerns (cons) based on the known cardiopulmonary effects of ultrafine particles (UFPs). We also discuss a limited number of studies using NPs on cellular and animal pulmonary toxicity and translocation to extrapulmonary sites. We selected the pro and con articles from the large body of literature on the basis of the number of subjects involved in epidemiologic studies and the consistency of reported studies. Studies on pro selections were based on the potential importance in biological or medicinal applications.

## UFPs versus NPs

UFPs and NPs, whether anthropogenic or engineered, are similar in size with diameters < 100 nm and possess many similar characteristics. The term “UFPs” traditionally has been used to describe airborne particles with diameters < 100 nm. The term “ultrafine” is frequently used to describe nanometer-size particles that have not been intentionally produced but are the incidental products of processes involving industrial, combustion, welding, automobile, diesel, soil, and volcanic activities. The ambient particulate matter (PM) produced from these sources contains particles in three sizes: < 0.1, 0.1–2.5, and > 2.5 μm. Most of the particle mass in the ultrafine size range is < 2.5 μm (PM_2.5_), with the largest number of particles < 0.1 μm (Hinds 1999). Hinds (1999) found that UFPs have longer lifetimes in the atmosphere, can be transported over thousands of kilometers, and remain suspended in air for several days. Furthermore, UFPs with greater surface area can carry large amounts of adsorbed pollutants, oxidant gases, organic compounds, and transition metals (Oberdörster 2001). The greater pulmonary deposition efficiency of UFPs with larger surface area and transition metals bound to them is considered important in cardiopulmonary toxicity.

In urban industrial locations, at least half the PM_10_ (PM < 10 μm in diameter) mass generated consists of PM_2.5_ with an average mean value of 13.4 μg/m^3^ (Dominici et al. 2006). UFPs possess a wide range of morphologic, chemical, physical, and thermodynamic properties. The UFPs emitted from different sources and geographic locations vary considerably in the types and concentrations of metal contaminants and aromatic compounds bound to surface. The primary particles emitted from the sources interact through chemical reactions in the atmosphere with oxygen, nitrogen dioxide, ozone, sulfur dioxide, and organics producing secondary particles of diverse reactivity and characteristics. The surface properties of UFPs generated at different sources and during aging of the particles are dynamically different in toxicity. Therefore, the toxicity and adverse health effects caused by UFPs are heterogeneous, depending on the source and mixed exposures of primary and secondary UFPs.

“NPs,” which in general terms are defined as engineered structures with diameters of < 100 nm, are devices and systems produced by chemical and/or physical processes having specific properties not displayed in their macro-scale counterparts. Milling or grinding may also produce NPs that may or may not have properties different from the bulk materials (National Nanotechnology Initiative 2005; U.S. Environmental Protection Agency 2004). The term “NPs” in this review is used, therefore, to differentiate engineered particles with a diameter of < 100 nm that are different from incidental UFPs.

## Pros: Applications in Biology and Medicine

### Imaging and diagnosis

Molecular imaging is an important discipline in biology and medicine with ability to detect, quantify, and display molecular and cellular changes that happen *in vitro* and *in vivo*. Fluorescent biological probes are used conventionally in biology because of their inert qualities and their ability to interact without loss of sensitivity in a variety of cellular reactions. However, there are intrinsic limitations with several organic dyes. The dynamic range of NPs, with diameters of < 100 nm, as probes attached to molecules of peptides, antibodies, or nucleic acids for the detection of cellular reaction products makes them ideal tools for display and quantification of molecular reactions *in vivo*. Such NP-based probes have high levels of brightness, photostability, and absorption coefficients across a wide spectral range (Niemeyer 2001). Their abilities to monitor ultrastructural interactions on a continuum make them ideal for applications in biology and disease. Furthermore, the potential for coating the NPs with antibodies, collagen, and other micromolecules makes them biocompatible for detection and diagnosis.

**Figure 1:**
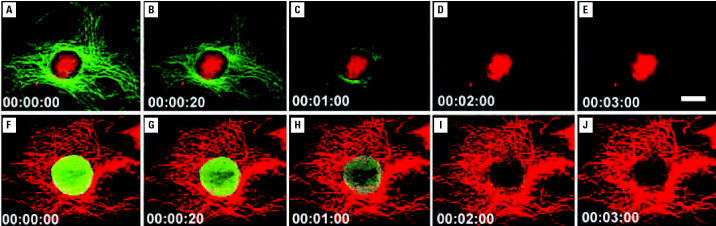
Fluorescent photostability and fluorescence intensity of quantum dots (QD 630) compared with organic dye Alexa 488. (*A–E*) Nuclei are labeled bright red with QD 630–streptavidin; actin fibers are stained green with Alexa 488. (*F–I*) Images of actin fibers are labeled red with QD 630–streptavidin; nuclei are labeled green with Alexa 488. Numbers in the bottom left corner indicate elapsed time. Scale bar, 10 μm. From Wu et al. (2003) and reproduced with permission from Quantum Dot Corp. (Hayward, CA).

**Figure 2:**
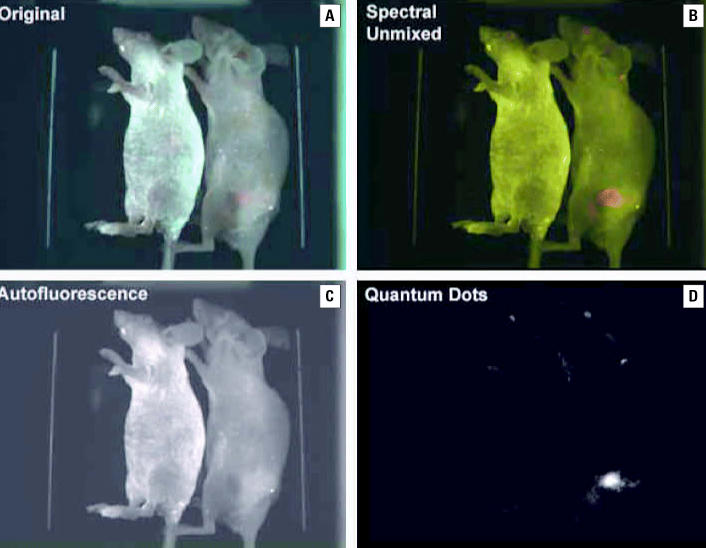
(*A,B*) Spectral images of quantum dot–prostate-specific membrane antigen conjugates in live animals with and without tumor (control). (*A*) Image of control animals, with no fluorescence (unmixed spectral). (*B*) Xenograft tumor-bearing animal showing bright red fluorescence of tumor. (*C*) Autofluorescent superimposed image of control and tumor-bearing animals. (*D*) Autofluorescent unmixed quantum dot image. From Gao et al. (2004) and reproduced with permission from the Nature Publishing Group.

An increasing number of studies in diagnosis and detection have been published, and we describe a select few here. In a study using mouse fibroblasts, Bruchez et al. (1998) showed that NP-based fluorescent labeling was better than conventional fluorophores. Wu et al. (2003) observed that quantum dot–based immunofluorescent labeling of the cancer marker Her2 was more efficient than conventional fluorophores in labeling different target cell–surface receptors, cytoskeleton, nuclear antigens, and other intracellular organelles. They also demonstrated that bioconjugated colloidal quantum dots were valuable in cell labeling, cell tracking, DNA detection, and *in vivo* imaging (Figure 1). Zhang et al. (2002) showed tht surface modification of superparamagnetite NPs with ethylene glycol and folic acid was effective in facilitating phagocytosis by cancer cells for potential cancer therapy and diagnosis. Gao et al. (2004) reported imaging and cancer targeting based on semiconductor quantum dots in animal studies *in vivo*. In control studies Gao et al. observed the uptake, retention, and distribution of quantum dots primarily in the liver, spleen, brain, heart, kidney, and lung in decreasing order. In nude mice growing human prostate cancer xenograft, quantum dots accumulated specifically at cancer targets showing bright orange red color (Figure 2B).

### Drug delivery

Site-specific-targeted drug delivery is important in the therapeutic modulation of effective drug dose and disease control. Targeted encapsulated drug delivery using NPs is more effective for improved bioavailability, minimal side effects, decreased toxicity to other organs, and is less costly. NP-based drug delivery is feasible in hydrophobic and hydrophilic states through variable routes of administration, including oral, vascular, and inhalation.

In drug delivery, several approaches are currently being tested for better site-specific delivery of an effective dose using liposomes, polymeric micelles, dendrimeres, ceramic NPs, iron oxide, proteins, covalent binding, adsorption, conjugation, and encapsulation methods (Moghimi et al. 2005). Extended circulation of liposomes with entrapped doxorubicin was reported to be 300-fold more effective, with better pharmacokinetic ability than free doxorubicin in the treatment of Kaposi’s sarcoma and metastatic cancer (Allen and Cullis 2004; Gabizon et al. 2003). Kumar et al. (2004) reported that NP surfaces modified with cationic chitosan were efficient for drug delivery both *in vitro* and *in vivo*. Gelperina et al. (2005) reported that in chemotherapy treatment for tuberculosis, NP-based drug delivery improved drug bioavailability, reduced dose frequency, and overcame the nonadherence problem encountered in the control of tuberculosis epidemics.

### Anticancer therapy

Conventional anti-cancer treatments are nonspecific to target killing of tumor cells, may induce severe systemic toxicity, and produce drug resistant phenotypic growth. An exciting potential use of nanotechnology in cancer treatments is the exploration of tumor-specific thermal scalpels to heat and burn tumors. O’Neal et al. (2004) observed in mice that selective photothermal ablation of tumor using near infrared-absorbing polyethylene-coated gold nanoshells of 130 nm inhibited tumor growth and enhanced survival of animals for up to 90 days compared with controls. Perkel (2004) also reported that antibody-coated magnetic iron NPs were effective to heat and literally cook the tumors. In similar work performed in athymic mice using antibody-coated iron NPs, DeNardo et al. (2005) showed specific targeted binding to tumors and tumor necrosis within 24 hr after therapy with better response. The efficacy of different antibodies conjugated to NPs, including transferrin and epidermal growth factor receptor, was examined in animal studies (DeNardo et al. 2005; El-Sayed et al. 2006). In cancer therapy, enzyme-mediated liposome destabilization and specific phospholipase A_2_ activation with synergistic membrane perturbing and permeability were reported to be more effective (Andresen et al. 2004).

### Gene therapy

Attempts to cure genetic diseases by transfer of somatic cells transfected with normal genes gained popularity in the last two decades. In gene therapy a normal gene is inserted in place of an abnormal disease-causing gene using a carrier molecule. Conventional uses of viral vectors are associated with adverse immunologic, inflammatory reactions, and diseases in the host. In this regard Gopalan et al. (2004) found NP-based gene therapy to be effective in systemic gene treatment of lung cancer using a novel tumor suppressor gene, FUS1. Chitosan, a polymer long used in gene therapy, was reported to have increased transfection efficiency and decreased cytotoxicity (Mansouri et al. 2006). Oral gene delivery in BALB/C mice using poly-l-lysine modified silica NPs has shown success with the distribution of particles throughout the intestinal mucous cells with limited cytotoxicity (Li et al. 2005b). Dufes et al. (2005) reported gene therapy by intravenous administration of NP-based vector systems using tumor necrosis factor (TNF)-α expression plasmid and found increased transgene expression and long-term survival of rats with no toxicity. Kaul and Amiji (2005) observed that tumor-targeted gene delivery using polyethylene glycol–modified gelatin NPs was highly effective, biocompatible, biodegradable, and long circulating for systemic delivery to solid tumors. Recent *in vitro* work with breast cancer cells has shown the potential efficacy of NP-mediated gene delivery of the wild-type *p53* gene. Cancer cells exposed to these NPs-based gene delivery showed an increased and sustained antiproliferative activity not seen in cells exposed to vector alone (Prabha and Labhasetwar 2004). Bharali et al. (2005) reported that a nonviral vector for *in vivo* gene delivery and fluorescent visualization of transfection using organically modified silica NPs has promising success for targeted brain therapy. The efficacy of NP-based transfection exceeded viral vector-based gene delivery, and *in vivo* optical imaging provided efficient and continual monitoring, retention, and viability of transfected cells.

From these studies, it is apparent that nanotechnology will profoundly affect human health through advances in medicine, science, and industry. The potential human benefits of nanotechnology are innumerable and include many aspects of human life with wide a variety of products. Few additional positive applications of NPs are listed in Supplemental Material, Table 1, available online (http://www.ehponline.org/members/2006/8871/supplemental.pdf).

## Cons: Morbidity and Mortality Due to Cardiovascular Effects

### Caveat

Since the beginning of the Industrial Revolution, anthropogenic sources of human exposure have increased dramatically, and based on a temporal correlation, the high concentrations of ambient air pollution and increases in morbidity and mortality were well established by several epidemiologic studies (Nel 2005). However, these epidemiologic data are not supported by a direct cause and effect relationship.

Numerous epidemiologic investigations have shown a direct credible relationship between ambient air particulate pollution and a consistent association with increased health effects specifically attributed to cardiovascular diseases. During the last few decades there has been a continued increase in the morbidity and mortality among adults and susceptible populations attributed to air pollution in industrialized and developing countries. The concentration–response relationship between PM_2.5_ and daily deaths has been reported to cause 100,000 deaths annually in the United States (Schwartz et al. 2002). In a recent comprehensive review of epidemiologic studies, Delfino et al. (2005) showed clearly the pathophysiologic changes associated with exposure to UFPs—changes that induce cardiovascular diseases.

A strong association of ambient particulate air pollution as a predictor of mortality and morbidity of adults in six polluted and less polluted U.S. cities was well documented in early two epidemiologic studies (Dockery et al. 1993; Pope et al. 1995). In a subsequent study the fine particulate burden was further linked to increased cardiovascular mortality and morbidity with physiologic correlates (Pope et al. 1999). In this study an increased heart rate was reported to be associated with increased exposure to airborne ambient particulates. Exposure to ambient air pollution was also shown to be associated with an increase in blood pressure and decreased heart rate variability with no apparent changes in oxygen saturation (Gold et al. 1998; Shy et al. 1998). Peters et al. (2000, 2001) showed that elevated levels of air pollution are associated with increasing incidence of life-threatening arrhythmia and triggering of myocardial infarction. They also showed that exposure to increased levels of air pollution for short durations of ≥ 2 hr triggered myocardial infarction. In a fine particulate air pollution and mortality study in 20 U.S. cities, a 0.68% increase in relative death rate from cardiovascular and respiratory causes was reported for each increase in the PM10 level of micrograms per cubic meter (Samet et al. 2000). Epidemiologic and pathophysiologic evidence supported the link of fine particulate air pollution to cause-specific cardiovascular mortality to diseases such as pulmonary and systemic inflammation, accelerated atherosclerosis, and altered cardiac autonomic function (Pope et al. 2004). In a 16-year followup of 500,000 adults in the Cancer Prevention Study II, Dockery et al. (2005) reported that a 10 μg/m^3^ increase in PM_2.5_ was associated with an 8–18% increase in mortality due to ischemic heart disease, dysrhythmias, heart failure, and cardiac arrest. Exposure for a short period of 3 days to increased ambient air pollution was also reported as a major factor for the increased disease outcomes (Dockery et al. 2005). In recently published population-based studies, it was also reported that short-term exposures to ambient PM_2.5_ and O_3_ are associated with cardiac autonomic dysfunction in older adults who had histories of cardiac and other diseases, showing an effect modification (Dockery et al. 2005; Park et al. 2005). Regional differences associated with PM_2.5_ and cardiovascular respiratory risks in the eastern, northwestern, and southwestern United States compared with other regions of the country were also reported recently (Dominici et al. 2006). The potential for UFPs to exacerbate preexisting cardiovascular risks is implicated in this effect. Peters et al. (1997) in a retrospective study on plasma viscosity during an air pollution episode reported an increase suggestive of a link to mortality.

The hypothesis that translocation of inhaled UFPs directly to systemic circulation causes a direct cardiovascular effect in individuals at increased risk was tested in a study on healthy subjects. In this study inhaled ultra-fine carbon particles labeled with technetium (Tc) were shown to pass rapidly into the systemic circulation of healthy male nonsmokers (Nemmar et al. 2002a). However, this finding has been refuted by several other studies as a methodologic overestimation of soluble ^99^Tc pertechnetate (Brown et al. 2002; Kreyling et al. 2002; Oberdörster et al. 2002). In a recent study Mills et al. (2006) showed conclusively that the majority of ^99^Tc-labeled carbon nanoparticles remained in the lung for up to 6 hr after inhalation.

Three mechanisms have been proposed to explain the events that may lead to cardio-pulmonary morbidity and mortality in populations exposed to fine ambient air pollution. The first hypothesis is that the fine PM are able to stimulate the neurons in the lung affecting the central nervous system and cardiovascular autonomic function. The second proposes that inhaled fine PM gains direct access to the systemic circulation and reaches target organs, thereby triggering inflammation, secretion of cytokines, reactive oxygen species (ROS), C-reactive proteins, and inducing cardiac events. The third hypothesis proposes that inhaled fine PM triggers an acute inflammatory response in the lung, thus stimulating the secretion of cytokines, chemokines, ROS, and transcription factors. The cascade of subsequent events and inflammation plays a key role in the activation of mitogen-activating protein kinase (MAPK), redox-sensitive transcription factors, nuclear factor kappa B (NF-κB), and activating protein-1 (AP-1), thereby promoting pulmonary inflammation that leads to cardiac events. There is a strong link supporting the relationship between inflammation and coronary heart disease because inflammation is associated directly with atherosclerosis (Sun et al. 2005). Furthermore, results from animal and cellular studies support credible mechanistic pathways induced by inflammatory responses that lead to systemic disease. In this respect, studies using genetically susceptible mice exposed to long-term air pollution PM showed acceleration of atherosclerosis and vascular inflammation, thereby supporting the indirect link between inflammation-induced developments of atherosclerosis (Sun et al. 2005). Evidence from human studies also supports this link to air pollution PM and development of cardiac responses that lead to atherosclerosis (Brook et al. 2004).

Several epidemiologic studies [see Delfino et al. (2005) and references therein] present the argument that ambient particles in the PM_2.5_ range can be correlated with adverse health effects. Characterization of human exposures to UFPs in these studies has been a limiting factor. Most of these studies used available indirect monitoring data to correlate adverse health effects without information on the proportions of UFPs or its specific toxic components. Consequently, criticisms have been raised about the possible nonspecific causal correlation for the observed associations. The lack of toxicologic evidence supporting these epidemiologic studies is answered partly by cellular and animal studies.

Animal experimental studies using concentrated ambient particles (CAPs) suggest that pulmonary vasculature is an important target for ambient air particle toxicity, as these particles exacerbate myocardial ischemia and induce coronary artery occlusion. Batalha et al. (2002) found that short-term exposure to CAPs induced vasoconstriction of small pulmonary arteries in normal rats and rats with chronic bronchitis. In dogs with coronary artery occlusion, Wellenius et al. (2003) observed that inhalation of CAPs resulted in the exacerbation of myocardial ischemia. The effect of UFPs in inducing experimental thrombosis in animal models supports the effect of particle size on the development of vascular thrombosis and pulmonary inflammations (Nemmar et al. 2002b). This ability of UFPs to evade phagocytosis and enter systemic circulation to reach extrapulmonary sites may be a relevant mechanism involved in cardiovascular mortality and morbidity, but it is unclear at this time. Seaton et al. (1995) hypothesized that *a*) UFPs reaching the cardiovascular system may induce coagulation, thrombosis, or other impairments or *b*) the persistent inflammation from UFPs in the lung promotes the release of mediators and cytokines, thus triggering cardiopulmonary events that lead to increased morbidity or mortality. In support of this hypothesis, Delfino et al. (2005) reported that patients with coronary heart disease had increased levels of inflammatory cytokines such as interleukin (IL)-1β, IL-6, TNF-α, C-reactive protein, and fibrinogen, compared with unaffected people.

This phenomenon was investigated by Nurkiewicz et al. (2004). Using an animal model, they showed the potential involvement of the systemic circulation caused by exposure to residual fly ash (ROFA < 2 μM), a surrogate for UFPs, or titanium dioxide (TiO_2_ < 1 μM) particles. They demonstrated that exposure to fine particles can impair systemic microvascular functional changes independent of any detectable pulmonary inflammation. Exposure to fine PM was associated with an influx of leukocytes in trapezius muscle venules of rats, demonstrating an endothelium-dependent arteriolar dilation and impairment. This was supported further by an increase in systemic blood pressure and a failure of microvessels to respond to intraluminal vasodilators (Nurkiewicz et al. 2004). These impairments may be the contributing factors involved in compromising the cardiovascular system, thereby leading to exacerbations and increased risk for heart attack in UFP-exposed populations. A recent study by Li et al. (2005a) showed that other mechanistic events induced by UFPs produced vasoconstriction by enhancing MAPK signaling via angiotensin type 1 receptor activation. Urban UFPs produced a time- and dose-dependent increase in phosphorylation of extracellular signal regulated kinase (ERK) 1/ERK2 and p38 MAPK. Copper and vanadium, two common metal contaminants in UFPs, also induced this activation of the local rennin–angiotensin system that plays an important role in cardiovascular effects. The water-soluble fraction containing copper and vanadium also induced phosphorylation of ERK1/ERK2 and p38 MAPK. *In vivo* support of these *in vitro* studies on the role of oxidative stress was demonstrated in an animal model compromised by pretreatment with dimethyl urea (Roberts et al. 2003). The molecular mechanisms promoted by ROFA-induced oxidative stress resulted in the activation of MAPK, inflammatory cytokines TNF-α and IL-6, and inflammatory protein MIP-2 (macrophage inflammatory protein 2) and provide insight into an inflammation-dependent triggering of events in the lung.

## Cons: Pulmonary Morbidity and Mortality

The lung is the major target of ambient air pollution and the relationship between increased ambient air pollution and adverse health effects in children, individuals with asthma, and vulnerable adults is well documented (Nel 2005). Particle size, surface area, and chemical composition all play a role in the health risks posed by PM. Increased respiratory symptoms, increased hospitalization, decreased lung function, increased respiratory infections, altered mucociliary clearance, chronic obstructive pulmonary disease (COPD), and increased mortality are some of the major documented adverse health effects caused by exposure to ambient air pollution (Gong et al. 2005; Koenig et al. 2005; Pietropaoli et al. 2004; Silkoff et al. 2005).

In provoking pulmonary health effects, exacerbation caused by pulmonary inflammation appears to play a major role. In susceptible individuals with asthma and COPD patients, exacerbation appears to be the important molecular mechanism by which UFPs exert their toxicity (Silkoff et al. 2005). Conversely, long-term health effects such as pneumoconiosis and cancer, which have subtle early detectable symptoms, remain difficult to establish because of the long latency of these diseases. Diseases exacerbated by acute inflammation such as asthma and COPD are well documented with corresponding fluctuations in ambient air pollution (Gong et al. 2005; Koenig et al. 2005; Pietropaoli et al. 2004; Silkoff et al. 2005). It is reasonable to expect that different molecular mechanisms may be involved in the genesis of cardiovascular and pulmonary diseases. Experimental studies have consistently documented that exposure to UFPs and NPs is more inflammatory to the lung, and a fraction of the inhaled UFPs is translocated to different extra-pulmonary sites of blood, liver, heart, spleen, and brain (Nemmar et al. 2003; Renwick et al. 2004). The extrapulmonary translocation is variable depending on particle size, chemical characteristics, and surface features. A recent study by Geiser et al. (2005) shows that inhaled TiO_2_ UFPs cross cellular membranes by non-phagocytic mechanisms in the lungs and were found in capillaries.

### Experimental cellular studies

Experimental studies using laboratory-generated UFPs and airborne CAPs have shown consistently higher pulmonary inflammatory and toxicity responses to UFPs (Brown et al. 2001; Dick et al. 2003; Donaldson et al. 2004a; Donaldson and Tran 2002; Saldiva et al. 2002). It is believed that UFPs provoke an increased oxidative stress because of their greater surface areas that allow them to interact with more cellular structures and different types of transition metals often associated with these particles (Dick et al. 2003; Donaldson et al. 2004b; Saldiva et al. 2002). The importance of the surface area within a narrow size range from 10 to 50 nm was recently demonstrated using acute lung inflammation as an end point after exposure to six different UFPs (Stoeger et al. 2006). Interactions between UFPs and associated transition metals were reported to have a synergistic mechanism in ROS generation and inflammation (Brown et al. 2001; Donaldson et al. 2004b). Differences in size and composition of UFPs compared with fine and larger particles were well correlated in studies of their uptake by different cell systems and their ability to induce oxidative stress (Brown et al. 2001; Dick et al. 2003; Kreyling et al. 2002). UFPs were shown to be the most potent inducers of oxidative stress in macrophages and epithelial cells by inducing heme oxygenase-1 and depleting intracellular glutathione. Oxidative stress induced by UFPs was also reported to be involved in the activation of MAPKs, which leads to the intracellular signaling of gene expression, and in the activation of AP-1 and NF-κB, which are important in the expression of proinflammatory genes and cytokines, including adhesion molecules (Oberdörster 2001; Oberdörster et al. 1995).

### Reactive oxygen species

*In vitro* and *in vivo* studies using CAPs and laboratory-made UFPs with different chemical composition and particle sizes have shown that ROS production is a major contributing factor in inflammation and toxicity. Investigators attribute the ability of UFPs to cause lung injury and disease to the larger surface area, smaller size, and metal contaminants (Donaldson et al. 2004a, 2005b; Nel et al. 2006; Oberdörster et al. 2005b). In addition NP-induced generation of ROS leading to oxidative stress, activation of signaling pathways, and apoptosis are considered new insights into the development of pulmonary and other diseases. This was illustrated in a hierarchical oxidative stress model that showed the correlation of oxidative stress and corresponding changes in cellular responses induced by UFPs (Nel et al. 2006)

Among the pathways implicated for the increased cardiopulmonary mortality and morbidity, the oxidant-dependent proinflammatory mechanisms are considered important based on *in vitro* and *in vivo* studies. The oxidative potential of UFPs collected from divergent sources such as natural dust, oil fly ash, coal fly ash, and ambient air was attributed primarily to their metal composition (Prahalad et al. 1999). Several metals identified in UFPs can generate ^•^OH radicals by Fenton-like reactions directly or after cellular reductions. It was also shown that both water-soluble and insoluble components of the UFPs have oxidant generation potential. In a study of healthy and pathogen-challenged animals, Antonini et al. (2002) observed that ROFA exposure resulted in severe lung damage and inflammation, thereby implicating altered oxidative stress in susceptible exposed animals.

### Pulmonary pathologic response

*In vivo* studies using CAPs or laboratory-generated UFPs also showed significantly greater inflammatory and toxic pulmonary responses in animals, depending on fine particle size and chemical composition. Oberdörster et al. (1994) observed that ultrafine TiO_2_ instilled into rats and mice was more proinflammatory than fine TiO_2_, as TiO_2_ induced a neutrophilic influx into the lungs. Oberdörster et al. (1994) reported that particle surface chemistry is equally or more important in inflammation and acute toxicity. Freshly generated polytetrafluethylene (PTFE) fume containing UFPs < 26 nm induced hemorrhagic pulmonary inflammation and death after rats were exposed to 10–30 μg of dust, and aging of UFPs resulted in the loss of surface reactivity and toxicity (Oberdörster et al. 1995).

Rats are often considered to be a sensitive and exaggerated-response animal model for particle-induced pulmonary investigations. Therefore, investigators in two recent independent studies used mice that were exposed to single-wall carbon nanotubes (SWCNT) and observed significant pulmonary pathologic changes with small as well as higher doses (Lam et al. 2004; Shvedova et al. 2005). In the study by Lam et al. (2004), all doses (3.3–16.6 mg/kg body weight) induced granulomatous lesions with persistent inflammation up to 90 days. Shvedova et al. (2005), using SWCNTs with minimal impurities at a dose of 10–40 μg/mouse, also found robust acute inflammatory response with the onset of pulmonary fibrosis associated with decreases in pulmonary function.

A comprehensive study conducted using the sensitive-response animal model (rat) in parallel with toxicity assessment showed controversial results. Rats exposed to SWCNT, silica, or carbonyl iron as positive and negative controls, respectively, showed surprisingly contradicting results (Warheit et al. 2004). They reported pulmonary granuloma formation in the absence of toxicity, inflammatory influx, and possible regression of granulomas over time. The spectrum of toxicologic responses that is so well documented with particulate exposure in several animals and humans is inconsistent and doubtful with respect to the data presented by Warheit et al. (2004) when one compares the dose to which animals were exposed per gram body weight. Lam et al. (2004) and Shvedova et al. (2005) exposed mice to doses smaller than those used by Warheit et al. (2004) in the sensitive animal model, and reported findings of multifocal granulomas of no physiologic significance. The relevance of this speculative interpretation is unfounded.

## Cons: Translocation and Toxicity to Other Organs

In the past, cardiovascular, neurologic, and excretory systems have not been considered secondary targets in inhalation toxicology and pathobiology. However, in recent years many animal and human studies have shown the translocation of UFPs to extrapulmonary sites such as systemic circulation, liver, heart, and brain (Kreyling et al. 2002; Nemmar et al. 2002b; Oberdörster et al. 2002). Although currently the process of UFP translocation is poorly understood, these preliminary studies provide consistent toxicologic backup for the hypothesis that UFPs are translocated to other organs, including heart, thus, playing a role in triggering and/or promoting cardiovascular morbidity and mortality. Several research studies involving UFPs have demonstrated the enhanced ability of these particles to penetrate more deeply into the lung interstitium than larger particles and evade clearance (Geiser et al. 2005; Oberdörster 2005a; Oberdörster and Utell 2002). This ability of UFPs to evade clearance promotes longer retention time in pulmonary interstitium, thus increasing the potential for translocation to extrapulmonary sites to exert effects. In an inhalation exposure study of rats, Oberdörster et al. (2002) reported that a significant amount of ^13^C accumulated in liver within 30 min postinhalation with a 5-fold increase in 1 day. They also observed that after inhalation, UFPs were transported to olfactory nerves at a speed of 2.5 mm/hr . From the available evidence, Hoet et al. (2004) concluded that phagocytosis by alveolar macrophages and endocytosis by the epithelial and endothelial cells are the important routes for translocation of UFPs to systemic circulation and then to other extra-pulmonary sites.

### Neuronal translocation

The potential for neuronal uptake and translocation of inhaled particulates and pathogens to the brain, which has been reported in several studies, was reviewed in detail by Oberdörster et al. (2005b). According to studies cited in their review, the olfactory nerve is the most viable pathway for the transport of particles intra-nasally inhaled because of the close proximity of the olfactory mucosa and bulb. In studies using whole-body inhalation exposure of rats to ultrafine carbon black, extrapulmonary translocation through the olfactory nerve was reported to be a viable mechanism (Oberdörster et al. 2002, 2004). Whether these NPs that are transported to the brain cause cell injury or toxicity to the brain is not known.

### Dermal exposure and translocation

The human skin is the largest organ in the body, protecting against the environment with a surface area of nearly 18,000 cm^2^. Polar and nonpolar materials can permeate across the stratum corneum via a paracellular route (Menon and Elias 1997). Photomechanical waves have been shown to enhance permeability of the stratum corneum *in vivo,* inducing expansion of the lacunar spaces, which leads to the formation of transient channels to facilitate the transport of macromolecules into the viable epidermis (Menon et al. 2003). A metabolic intervention to enhance effective transdermal drug delivery is reported to be highly effective in through permeabilized stratum corneum (Elias et al. 2002).

Penetration of particles > 1 μm is limited through healthy skin except in areas that have been scratched, injured, or mechanically stretched. In conjunction with physical activity, Tinkle et al. (2003) demonstrated in an animal model that topically applied 0.5- and 1.0-μm beryllium particles penetrate the stratum corneum and develop hapten-specific, cell-mediated immune response. Penetration of TiO_2_ microparticles contained in sunscreen into the stratum corneum and follicular orifice of hair has been reported (Lademann et al. 1999). Particles reaching the dermis can be transported to the lymphatic system by macrophages and dendritic cells. Although there are no well-documented studies on NP penetration and transmigration to other distant organs, one can assume from the results of reported studies using beryllium particles that it is more likely a viable route of entry in workers involved in mechanical or strenuous activities.

In a cytotoxicity study of human keratinocytes in culture, Shvedova et al. (2003) demonstrated the potential of SWCNT exposure to induce ROS generation, which results in cytotoxicity, lipid peroxidation, antioxidant depletion, and loss of cell viability associated with ultrastructural and pathologic changes. Their studies concluded that exposure to unrefined SWCNT can result in accelerated oxidative stress and toxic manifestations in workers.

**Figure 3:**
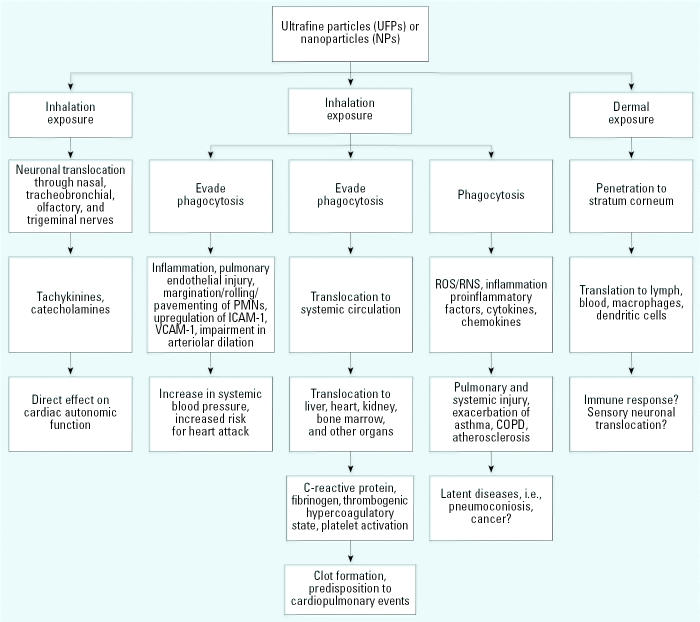
Hypothetical schema of potential interactions that may occur via inhalation of UFPs and translocation to other organs. Abbreviations: ICAM-1, intracellular adhesion molecule-1; PMNs, polymorpho-nuclear leukocytes; RNS, reactive nitrogen species; VCAM-1, vascular adhesion molecule-1. Schema also shows suspected interactions (indicated by a question mark) leading to sequences of events that may cause cardiovascular and pulmonary morbidity and mortality.

On the basis of current available toxicologic studies and limited human data, we have developed a schema of potential interactions of UFP transportation, and suspected sequence of events that may lead to cardiovascular, pulmonary and other organ involvement (Figure 3).

## Conclusions

Advances in nanomedicine offer the possibility of new and intriguing opportunities in NP-based early detection, diagnosis, and treatment of diseases. Commercial development of nanotechnology and its many applications may unleash a spectrum of human health hazards that at the present time can only be speculated about without any detailed understanding of the toxic nature of NPs. The review of literature in the fields of toxicology and the possible human health effects of UFPs and NPs provide only a glimpse of some toxic paradigms that compel us to weigh the adverse effects against the beneficial effects. Because we know little about the toxic health hazards of NPs *in vivo* and *in vitro,* pharmacokinetic and toxicologic studies are mandatory before large-scale industrial production and use are implemented. In this regard the U.S. Environmental Protection Agency, the International Life Sciences Institute Research Foundation, and the Risk Science Institute convened working groups comprising experts in the fields of nanotechnology from academia and government to develop new toxicity screening, reporting, and hazard identification of engineered nanomaterials (Oberdörster et al. 2005a, 2005b). Although at this time, the benefits of nanotechnology dominate our thinking, the potential for undesirable human health outcomes should not be overlooked. Consistently large numbers of studies have reported associations between UFP exposure and morbidity in elderly and compromised individuals. Furthermore, recent studies also emphasize the impact of day-to-day variations in particle concentrations and exposures for short periods as important factors in cardiac events in predisposed population. Therefore, there is reason to suspect that NPs with size and surface characteristics similar to UFPs are likely to cause diseases—some with a long latency. With widespread industrialization of nanotechnology, there is the potential for ambient air pollution and a conceivable threat to the general population.

Doubtless, nanotechnology will have a profound impact on a wide range of applications and therefore on many aspects of human life, including environmental decontamination, water purification, cheaper electricity, and better disease treatment modalities. One major challenge facing industry and government is the lack of information on the possible adverse health effects caused by exposure to different nanomaterials. Development of safety guidelines by government for the nanotechnology industries, including manufacturing, monitoring of worker exposure, ambient release of NPs, and risk evaluations, is mandatory to promote nanotechnology for its economic incentives and medicinal applications.
