# Supplementary material for: Nanoparticles: Health Effects—Pros and Cons
Source: Environ Health Perspect. 2006 Aug 18;114(12):1818–25. doi: 10.1289/ehp.8871 (PMC1764161; doi:10.1289/ehp.8871)
Supplement: Supplemental Figures and Tables [file ehp0114-001818s1.pdf]

Supplemental Material. Examples of positive applications of engineered nanoparticles (NPs) in biology and medicine and limited number of studies on toxic potential

Reference	Application	Potential beneficial/ adverse effect	Pros/Cons
Alivisato Nature Biotechnology 22: 47, 2004	Biological detection	Sensing at the single molecule level	Pros
Kim et al. Nature Biotechnology 22 93, 2003	Imaging	Quantum dots - lymph node mapping	Pros
Bruchez et al. Science 281: 2013, 1998	Imaging/diagnosis	Fluorescent biological labels	Pros
Gao et al. Nat Biotechnol 22: 969, 2004	Imaging/diagnosis	In vivo cancer targeting/imaging	Pros
Wu et al. Nat Biotechnol 21: 41, 2003	Imaging/diagnosis	Quantum dots for cancer marker	Pros
Zhang et al. Biomaterials 23: 1553, 2002	Imaging/diagnosis	Magnetite NPs cellular uptake	Pros
Allen and Cullis Science 303: 1818, 2004	Drug delivery	Lipid or polymer-based NPs improve drug delivery	Pros
Andresen et al.J Med Chem 47: 1694, 2004	Drug delivery	Liposome-based chemotherapeutic drugs	Pros
Banerjee et al. Internation J Pharma 243: 93, 2002	Drug delivery	Chitosan NPs biodistribution	Pros
Gabizon et al.Clin Pharmacokinetic 42: 419, 2003	Drug delivery	Pegylated liposomal drug use	Pros
Gelperina et al. Am J Respir Crit Care Med 172 (12): 1487-90, 2005	Drug delivery	Chemotherapy of tuberculosis	Pros
Giri et al.Med Materials Eng 13: 387, 2003	Drug delivery	Hyperthermia - killing of tumor cells	Pros
Kozower et al.Nat Biotech 21: 392, 2003	Drug delivery	Immunonotargeting catalase	Pros
Kumar et al.J Nanosci Nanotechnol 4: 990, 2004	Drug delivery	Gene transfection agents	Pros
Moghimi et al.FASEB J 19: 311, 2005	Drug delivery	Applications of nanotechnology	Pros
Serpe et al.Euro J Pharmaceutics Biopharmaceutics 58: 673, 2004	Drug delivery	Solid lipid NPs to minimize cytotoxicity of conventional drugs	Pros
DeNardo et al.Clin Cancer Res 11: 7087, 2005	Cancer therapy	Tumor targeting cancer therapy	Pros
Dobson J. Gene Ther 13: 283, 2006	Cancer therapy	Magnetic NPs-based gene delivery	Pros
El-Sayed et al. Cancer Lett 239 (1): 129-35, 2006	Cancer therapy	Photo-thermal therapy for cancer	Pros
Kommareddy et al. Technol Cancer Res Treat 4: 615, 2005	Cancer therapy	Tumor-selective gene delivery	Pros
Mortensen et al. Appl Radat Isot 64: 315, 2006	Cancer therapy	T-cell-guided melanoma irradiation	Pros
Mossman et al.Vaccine 23: 3545, 2005	Cancer therapy	Development of NPs-based vaccine	Pros
O'Neal et al.Cancer Lett 209: 171, 2004	Cancer therapy	Photothermal tumor ablation	Pros
Perkel The Scientist August 30, 14, 2004	Cancer therapy	Health	Pros
Weissleder et al. Nature Biotechnology 23: 1418,2005	Cancer therapy	Cell-specific targeting by NPs	Pros

Bharali et al.PNAS 102: 11539, 2005	Gene therapy	Silica NPs-gene delivery to brain	Pros
Brannon-P and Blanchette Adv Drug Deliv Rev 56: 1649, 2004	Gene therapy	NP targeted cancer therapy	Pros
Dufes et al.Cancer Res 65: 8079, 2005	Gene therapy	Synthetic anticancer gene therapy	Pros
Gopalan et al.Technol Cancer Res Treat 3: 647, 2004	Gene therapy	NPs-based gene therapy,suppress inflammatory response	Pros
Kaul and Amiji Phar Res 22: 951, 2005	Gene therapy	PEG modified NPs - gene elivery	Pros
Li et al.J Nanosci Nanotechnol 5: 1199, 2005	Gene therapy	Oral gene therapy	Pros
Luo and Saltzman, Nature Biotechnol 18: 893, 2000	Gene therapy	Silica NPs enhanced transfection	Pros
Mansouri et al.Biomaterials 27(9):2060-5, 2005	Gene therapy	Folate-chitosan-DNA gene therapy	Pros
Prabha and Labhasetwar Mol Pharm 1: 211, 2004	Gene Therapy	p53 gene delivery-breast cancer	Pros
Wen et al. Pest Manag Sci 61: 583, 2005	Pesticide carriers	Controlled release of avermectin	Pros
Nair et al. J Environ Monit 5: 363, 2003	Pesticide detection	Endosulfan detection by gold NPs	Pros
Crespilho et al. Environ Sci Technol 39: 5385, 2005	Pesticide sensor	Humic acid in NP layered film-PCP	Pros
Liu and Lin Anal Chem 77: 5894, 2005	Pesticide sensor	Zirconia NPs-organophosphate sensor	Pros
Quan et al. Environ Sci Technol 39: 3770, 2005	Environment	Titania nanotubes for PCP	Pros
Klupinski et al. Environ Sci Technol 38: 4353, 2004	Fungicide removal	Iron oxide NPs induced reduction	Pros
Dingler et al.J Microencapsulation 16: 751, 1999	Health	Lipid NPs dermal application	Pros
Mills et al Am J Respir Crit Care Med 173: 426, 2006	Human	NPs no systemic translocation	Pros
Ellsworth et al. Chemical Innovation 30: 30-35, 2000	Industry	Environment	Pros
Jennings et al. Eur J of Pharma and Biopharma 49: 211-8, 2000	Industry	Environment	Pros
Varghese and Grimes J Nanosci Nanotech 3: 277, 2003	Industry	Metal oxide NPs for environmental sensing	Pros
Salata J Nanotechnology 2: 3, 2004	NPs applications	Recent developments review	Pros
Oberdörster et al. Environ Health Perspect 113 (7): a488	Human/animal	Nanotoxicology	Pros/Cons
Delfino et al. Environ Health Perspect 113:934, 2005	Human	Cardiovascular health-Review	Cons
Kreyling et al. J Toxicol Environ Health 65: 1513, 2002	Human	Translocation	Cons
Nemmar et al.Circulation 105: 411, 2002	Human	Translocation of NPs to blood	Cons

Hoet et al.J Nanotechnology 2:12, 2004	Human/animal	NPs known and unknown health risks	Cons
Gurr et al. Toxicology 213: 66, 2005	Human/cellular	Titanium NPs induce oxidative damage	Cons
Peters et al. J Material Sci 15: 321, 2005	Human/cellular	Endothelial cell function, inflammation, proliferation	Cons
Brown et al. Am Rev Respir Crit Care Med 166: 1240, 2002	Human/disease	Patients with obstructive disease receive increased dose of ultrafine particles	Cons
Renwick et al. Occup Environ Med 61: 442, 2004	Animal	Increased inflammation and chemotactic response by NPs	Cons
Meiring et al.Particle Fibre Toxicol 2: 3, 2005	Animal	Translocation of iridium NPs	Cons
Oberdörster et al. J Toxcol Environ Health 65: 1531, 2002	Animal	Carbon NPs translocation to blood, liver	Cons
Geiser et al.Environ Health Perspect 113: 1555, 2005	Animal/cellular	Translocation of titanium NPs by nonphagocytic mechanisms	Cons
Lademann et al.Skin Pharmacol Appl Skin Physiol 12:247-56, 1999	Animal/cellular	Translocation of titanium in skin	Cons
Lam et al. Toxicol Sci 77: 126-34, 2004	Animal/cellular	Pulmonary fibrosis effects of carbon NPs	Cons
Nemmar et al. Am J Respir Crit Care Med 166: 998, 2002	Animal/cellular	Experimental thrombosis induced by NPs	Cons
Shvedova et al. Am J Physiol Lung Cell Mol Physiol 289(5):L698-708, 2005	Animal/cellular	Pulmonary fibrosis response of carbon NPs	Cons
Warheit et al. Toxicol Sci 77: 117-25, 2004	Animal/cellular	Pulmonary fibrosis effects of carbon NPs	Cons
Geys et al.Toxicol Lett 160: 218, 2006	Cellular	Pulmonary translocation	Cons
Wottrich et al. Int J Hyg Environ Health 207(4) 353-61, 2004	Cellular	Hematite/silicasol uptake biological effects	Cons
